# Bioprinting collagenase-responsive hydrogel for controlled release of cowpea mosaic virus immunotherapy

**DOI:** 10.1088/1758-5090/ae37de

**Published:** 2026-01-28

**Authors:** Zhongchao Zhao, Yi Xiang, Zhuohong Wu, Yazhi Sun, Jacob Schimelman, Steven Fiering, Shaochen Chen, Nicole F Steinmetz

**Affiliations:** 1 Aiiso Yufeng Li Family Department of Chemical and Nano Engineering, University of California, San Diego, 9500 Gilman Dr, La Jolla, CA 92093, United States of America; 2 Center for Nano-ImmunoEngineering, University of California, San Diego, 9500 Gilman Dr, La Jolla, CA 92093, United States of America; 3 Moores Cancer Center, University of California, San Diego, 9500 Gilman Dr, La Jolla, CA 92093, United States of America; 4 Department of Radiology, University of California, San Diego, 9500 Gilman Dr, La Jolla, CA 92093, United States of America; 5 Department of Microbiology and Immunology, Geisel School of Medicine at Dartmouth, Lebanon, NH 03756, United States of America; 6 Dartmouth Cancer Center, Geisel School of Medicine at Dartmouth, Lebanon, NH 03756, United States of America; 7 Department of Bioengineering, University of California, San Diego, 9500 Gilman Dr, La Jolla, CA 92093, United States of America; 8 Institute for Materials Discovery and Design, University of California, San Diego, 9500 Gilman Dr, La Jolla, CA 92093, United States of America; 9 Center for Engineering in Cancer, University of California, San Diego, 9500 Gilman Dr, La Jolla, CA 92093, United States of America; 10 Shu and K.C. Chien and Peter Farrell Collaboratory, University of California, San Diego, 9500 Gilman Dr, La Jolla, CA 92093, United States of America; 11 Department of Chemical Engineering, University of California, Davis, 1 Shields Ave, Davis, CA 95616, United States of America

**Keywords:** bioprinting, controlled release, hydrogel, immunotherapy

## Abstract

In this work, we developed a collagenase-responsive hydrogel system to covalently load cancer immunotherapy candidate cowpea mosaic virus (CPMV) using 3D digital light processing bioprinting technology. CPMV was functionalized with norbornene groups (CPMV-NB), which was then bioprinted into hydrogels with 8-arm polyethylene glycol norbornene and a collagenase-cleavable peptide via photoinduced thiol-ene click chemistry. This strategy enabled stable retention of CPMV-NB within the hydrogels and achieved controlled release of CPMV-NB triggered by collagenase. Furthermore, released CPMV-NB retained its immunogenicity to stimulate immune cells.

## 1. Main

Cowpea mosaic virus (CPMV) is a ∼30 nm plant virus [1]. It has a bipartite ssRNA genome encapsulated in its viral capsid which is composed of 60 copies each of a large (L) and small (S) coat protein [2]. Though non-infectious toward mammals [3],CPMV shows exceptional potency as an immunoadjuvant to stimulate innate and adaptive immune response enabling applications for cancer treatment [4–9]. Its anti-tumor efficacy has been reported in various murine tumor models including melanoma, breast cancer, colorectal cancer, glioma, and ovarian cancer, as well as in companion dogs with oral melanoma and mammary tumors [6, 8–12]. Mechanistic studies reveal that CPMV is recognized as a pathogen-associated molecular pattern to stimulate innate immune cells [13–21]. Specifically, its viral capsid functions as dual Toll-like receptor (TLR) 2 and TLR4 agonists and its RNA genome as a TLR7 agonist [4, 5]. When CPMV is administered intratumorally, it converts the immunosuppressive tumor microenvironment (TME) into an anti-tumor TME, priming innate immune cell activation, which leads to tumor cell killing and establishment of long-lasting adaptive anti-tumor immunity [8, 9]. All these findings highlight CPMV as a promising cancer immunotherapy candidate for clinical translation in human disease treatment [22].

As with most cancer (immuno)therapies, CPMV treatment requires repeated dosing to achieve its therapeutic outcome, i.e., local and distant tumor regression and prevention of recurrence. While weekly dosing for cutaneous tumors is clinically feasible, repeat dosing for non-cutaneous tumors such as pancreatic cancer, brain tumors, or ovarian cancer may be more challenging requiring interventional radiology tools (e.g. ultrasound-guided administration) and may require hospitalization leading to decreased quality of life for patients and increased financial burden. To overcome this barrier, developing an approach that enables controlled and sustained release of CPMV will alleviate the need for repetitive dosing and the burden from hospitalization.

Various strategies have been reported to achieve controlled release of CPMV, including hot melt extrusion [23],microneedle patches [24],dendrimer co-assembly [25],and nanocapsules [26]. However, these strategies heavily relied on the passive diffusion of CPMV from the formulated materials. In our previous work [27],we explored 3D digital light processing (DLP) bioprinting to fabricate a CPMV-loaded hydrogel depot, which was surgically implanted into the intraperitoneal (IP) space of mice and used as a slow-release formulation to treat IP-disseminated ovarian tumors. This approach enabled slow release of CPMV and improved treatment efficacy after single administration in a murine ID8-Defb29/Vegf-a-Luc ovarian cancer model. However, our study also showed that the CPMV-hydrogel was fully dissolved leading to complete CPMV release within 4 weeks. Given the slowly-progressing ovarian tumors, we previously defined that a 6+ week treatment window is most effective to achieved potent efficacy against the murine ID8-Defb29/Vegf-a-Luc ovarian tumor model [9]. Thus, there is a potential gap between our previous CPMV hydrogel’s release duration and the reported therapeutic window required for optimal CPMV treatment outcome. Therefore, in this work, our goal was to further improve our CPMV hydrogel system to support sustained release throughout the full therapeutic timeframe–from initial tumor establishment to the typical disease outburst period–to achieve maximal suppression and treatment of ovarian cancer. To accomplish this, we sought to engineer our CPMV hydrogel system into an enzyme-responsive release platform, enabling controlled and enzyme-triggered release of CPMV within the enzyme-enriched TME. Here we report a proof of concept (POC).

We modified our previously explored bioink formulations by replacing gelatin methacrylate and polyethylene glycol diacrylate with 8-arm PEG norbornene (PEGNB) and a matrix metalloprotease (MMP) cleavable peptide crosslinker KC-VPMSMRGG-CK (SH) [28–30]. This peptide contains two cysteine residues, which offer thiol groups for photoinduced thiol-ene off-stoichiometric crosslinking to form the hydrogel network during the DLP bioprinting process [28–30]. The crosslinker also doubles as a cleavable bioconjugation site for the norbornene-functionalized CPMV nanoparticle, enabling covalent encapsulation of CPMV and its controlled release upon MMP-mediated cleavage of the peptide sequence.

**Figure 1.**
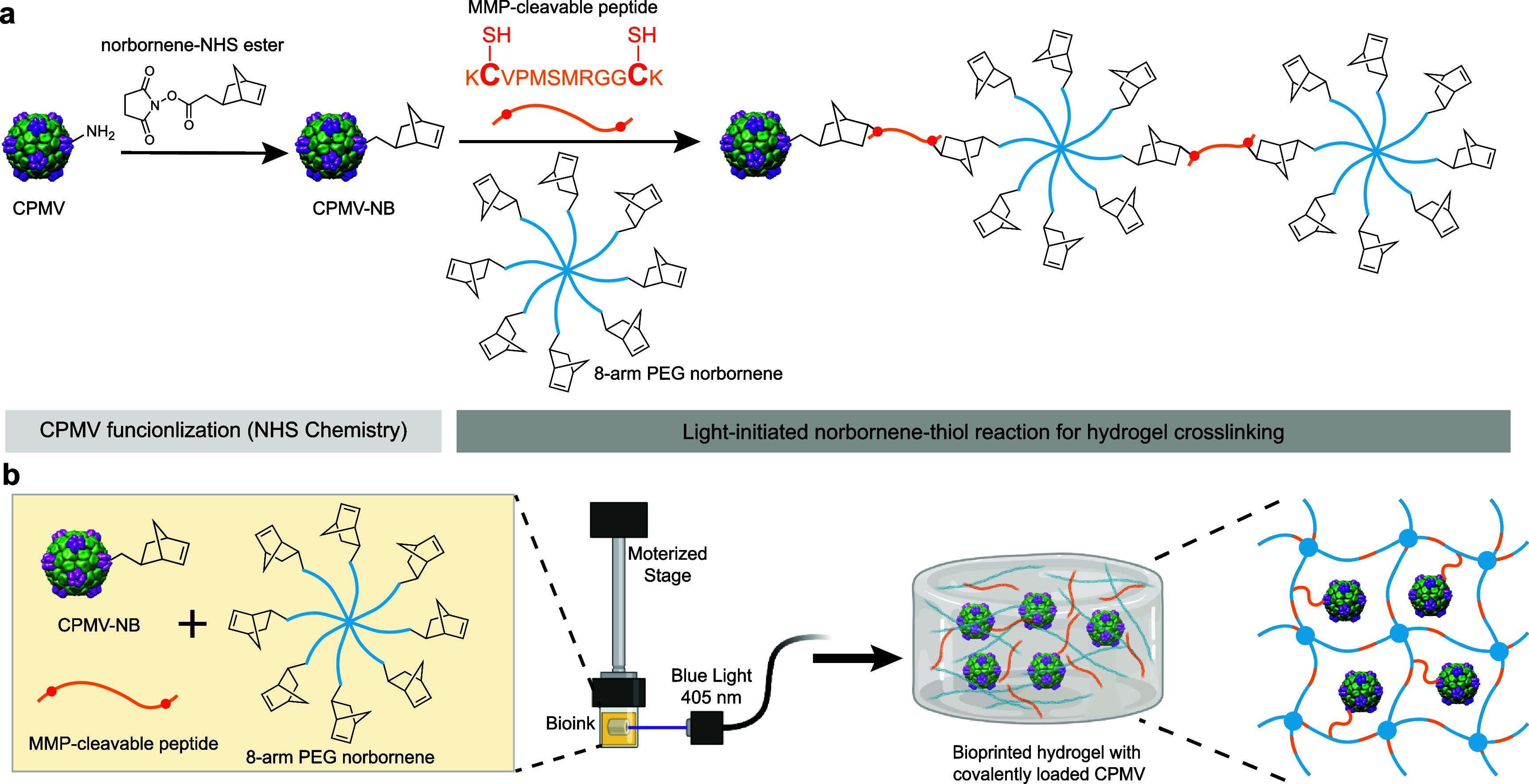
Bioprinting CPMV-NB hydrogel. (a) CPMV is functionalized with norbornene-NHS ester via NHS chemistry to produce CPMV-NB, which is then crosslinked to 8-arm PEG norbornene via an MMP cleavable peptide. (b) Scheme of the CPMV-NB hydrogel printing process. CPMV-NB, MMP-cleavable peptide, and 8-arm PEG norbornene as components of the bioink are bioprinted into hydrogels through light-initiated thiol-norbornene reaction. Part of (b) was generated in BioRender. Created in BioRender. Steinmetz, N. (2026) https://BioRender.com/z9q330n.

CPMV was propagated in and purified from infected black eyed pea No. 5 plants [31, 32]. Each CPMV possesses 300 surface-exposed lysine residues, which enables functionalization via N-hydroxysuccinimide (NHS) chemistry [33]. To achieve coupling of CPMV with the MMP-cleavable peptide during the bioprinting process, CPMV was functionalized with norbornene groups (CPMV-NB) by reacting CPMV with 5-norbornene-2-acetic acid succinimidyl ester (norbornene NHS-ester) (figure 1(a)). The resulting CPMV-NB was then combined with PEGNB and KC-VPMSMRGG-CK as the bioink for 3D hydrogel bioprinting to achieve covalently trapping CPMV within the hydrogel network (figure 1).

Because our long-term objective is to develop an enzymatically triggered CPMV release platform for application as slow-release immunotherapy for non-cutaneous and slowly progressing tumors, we focused on optimizing our bioink formulations to maximize the CPMV retention within printed hydrogels. This optimization ensures that sufficient CPMV is entrapped for sustained, and enzyme-responsive release to enhance the therapeutic window. To achieve this, a series of CPMV-NB conjugates were synthesized by varying the molar ratio of CPMV to norbornene NHS-ester: CPMV-NB (1:120), CPMV-NB (1:600), CPMV-NB (1:1200), and CPMV-NB (1:2400). Denaturing gel electrophoresis (NuPAGE) showed both L (∼42 kDa) and S (∼24 kDa) proteins with molecular weights comparable to that of native CPMV coat proteins (supplementary figure 1(a))—this is as expected, the NB moiety with its molecular weight of only 249.26 Da is too small to show a mobility change in NuPAGE. Agarose gel electrophoresis confirmed that all conjugates remained intact as demonstrated by the co-migration of RNA genome (UV–Vis) and viral protein (Coomassie) (supplementary figure 1(b)). Furthermore, the slight increase in electrophoretic mobility toward the anode supports modification of Lys with NB, which leads to reduction of the positive surface charge contributed by the Lys side chains (the particles become more negative).

Different bioink formulations were then prepared with a constant CPMV-NB concentration, while varying PEGNB:SH ratios from 1:1 to 1:4. The bioinks were printed into 1 mm × 1 mm cylinders containing 10 *µ*g CPMV-NB using the DLP bioprinting system, in which a user-defined photomask was used to illuminate the bioink to initiate photopolymerization in the confined area, and a motorized stage was used to control the height of the printed structure [34]. Printed hydrogels were incubated in 1 ml phosphate-buffered solution (PBS) at 37 °C for 6 d to allow for diffusion of unbound CPMV-NB, followed by collagenase digestion. CPMV was quantified in supernatants of both diffusion and digestion respectively to identify conditions that retained CPMV-NB within hydrogels post diffusion by a CPMV ELISA assay. In the initial screening experiment, only three bioink formulations: CPMV-NB (1:600) PEGNB:SH (1:1), CPMV-NB (1:600) PEGNB:SH (1:2), and CPMV-NB (1:1200) PEGNB:SH (1:2) retained most CPMV-NB post diffusion (data not shown). Replication experiments confirmed that CPMV-NB (1:1200) PEGNB:SH (1:2) performed the best with maximal retention of CPMV-NB (supplementary figure 1(c)). Based on these results, this bioink formulation was selected for subsequent studies. Hereafter, CPMV-NB refers specifically to the CPMV-NB (1:1200) conjugate. Analysis by size exclusion chromatography confirmed that CPMV-NB remained intact after this processing showing the typical elution profile and co-elution of protein (280 nm) and RNA (260 nm) compared to CPMV (supplementary figure 1(d)).

**Figure 2.**
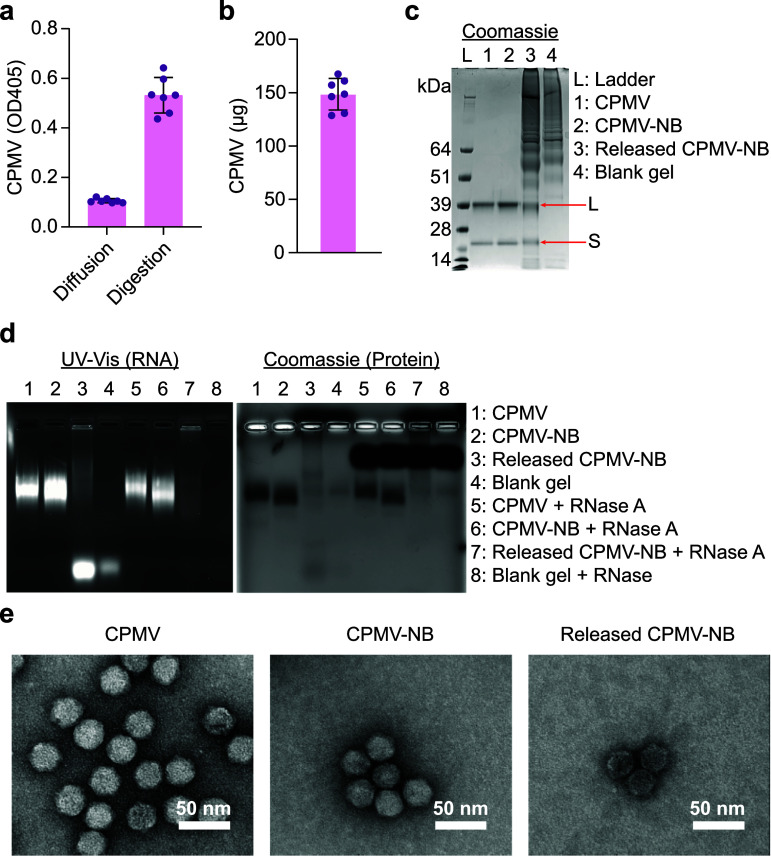
Characterization of CPMV, CPMV-NB, and released CPMV-NB from hydrogel. (a) CPMV ELSA to confirm that CPMV-NB is covalently attached within hydrogel. *n* = 7 independent experiments; data are expressed as mean ± SD. (b) Quantification of CPMV-NP within hydrogels by NuPAGE gel densitometry based on Supplementary figure 2. *N* =  7 independent experiments; data are expressed as mean ± SD. (c) NuPAGE of CPMV, CPMV-NB, released CPMV-NB after digestion, and blank hydrogel. All CPMV, CPMV-NB, and released CPMV-NB show the two CPMV subunit proteins, large (L) and small (S). (d) Agarose gel of CPMV, CPMV-NB, released CPMV-NB, and blank hydrogel with and without RNase A treatment. Gel is imaged by UV–Vis for RNA and by Coomassie for protein. (e) TEM images of CPMV, CPMV-NB, and released CPMV-NB from hydrogel.

Next, we bioprinted hydrogels into 5 mm × 5 mm × 1.5 mm slabs to load 200 *µ*g CPMV-NB for detailed characterization. Following diffusion and collagenase digestion, ELISA against CPMV showed that the majority of CPMV-NB was retained within hydrogel regardless of the hydrogel size (figure 2(a)). To accurately quantify the retained CPMV-NP in hydrogels, the digestion samples were analyzed by NuPAGE gel electrophoresis and gel densitometry, confirming ∼74% (∼148.4 *µ*g out of 200 *µ*g) of the loaded CPMV-NB was retained and ∼26% was released from the hydrogel (figure 2 (b) and supplementary figure 2). NuPAGE gel electrophoresis also showed that the released CPMV-NB exhibited L and S protein bands comparable to native CPMV and CPMV-NB (figure 2(c)), attesting to the high degree of stability of the assembled virions. Agarose gel electrophoresis of CPMV, CPMV-NB, and hydrogel-released CPMV-NB demonstrated electrophoretic mobility and co-migration of RNA and protein bands similar to that observed for native and unprocessed CPMV (figure 2(d), Lanes 1–3), indicating the CPMV, CPMV-NB, and hydrogel-released CPMV-NB samples remained mostly intact maintaining their packaged RNA cargos. However, the released CPMV-NB appeared in weaker signal with a less defined band and an additional high mobility bands (figure 2(d), Lane 3). It is possible that the concentration of this sample may have been underestimated for gel loading due to interference with digested hydrogel fragments. The band smearing also may be explained by interaction between CPMV-NB particles with digested hydrogel fragments. Lastly, strong UV–Vis signal was observed for released CPMV-NB (figure 2(d), Lane 3), indicating that CPMV-NB partially released its RNA due to enzyme digestion. To confirm this, all samples were treated with RNase (figure 2(d), Lane 5–8), demonstrating that the free RNA band could be removed prior to gel electrophoresis. Finally, transmission electron microscopy (TEM) confirmed that released CPMV-NB maintained its stable viral structure and morphology comparable to CPMV and freshly-prepared CPMV-NB (figure 2(e)). In conclusion, while subtle changes, such as some loss of RNA, was observed, overall the hydrogel-released CPMV particles retained a high level of structural integrity making this hydrogel delivery approach suitable for further efficacy testing.

**Figure 3.**
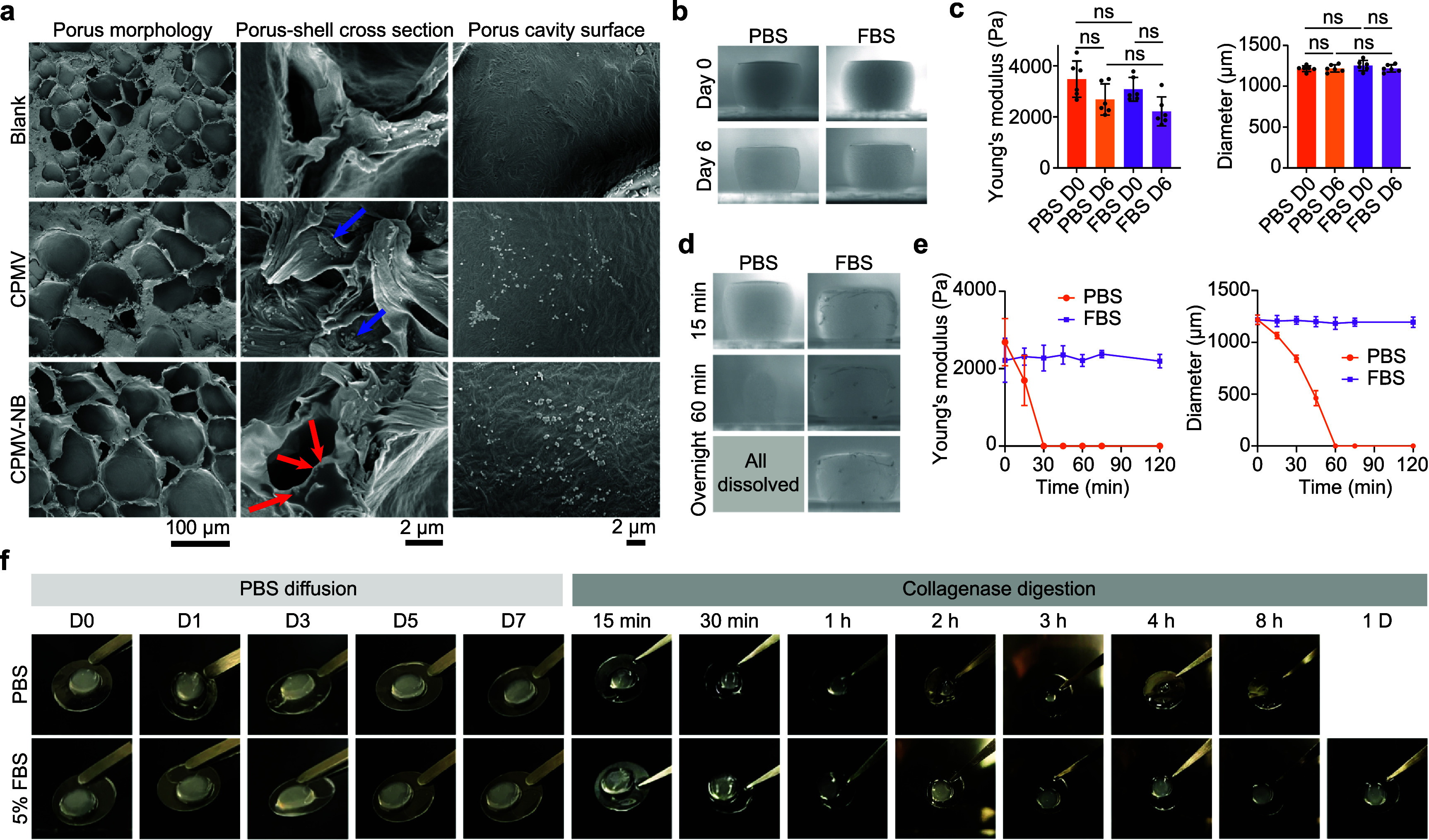
Characterization of bioprinted hydrogels. (a) SEM images of blank, CPMV, and CPMV-NB hydrogels. Red arrows: particles submerged in the matrix; Blue arrows: particles adhered to the surface of the matrix. (b) Images of hydrogels within PBS and FBS on Day 0 and Day 6. (c) Young’s modulus and diameter measurements of hydrogels in (b). *n* = 6 independent experiments; data are expressed as mean ± SD. (d) Images of hydrogels with collagenase treatment in PBS and FBS. (e) Young’s modulus and diameter measurements of hydrogels in (d). *n* = 6 independent experiments; data are expressed as mean ± SD. (f) Images of hydrogels in PBS and 5% FBS during the diffusion and collagenase digestion. Statistical significance was determined by ordinary one-way ANOVA.

The microstructure of the bioprinted CPMV-NB hydrogel was examined by scanning electron microscopy (SEM). As shown in figure 3(a), all hydrogel formulations exhibited a similar porous morphology at the bulk scale (left column). At higher magnification (middle column), regions containing CPMV in the CPMV-NB hydrogel displayed particles embedded or partially surrounded by the hydrogel matrix. As annotated by red arrows, the particles appear submerged, not touching a free surface. In contrast, in the blended hydrogel, CPMV particles were predominantly located on the surfaces of pore walls as aggregates, with their outline exposed (blue arrows). A similar trend was noted on the cavity surfaces, where the CPMV-NB hydrogel presented a greater number of surface-associated CPMV particles compared to the blended hydrogel.

These differences likely arise from the distinct incorporation mechanisms. In the CPMV-NB hydrogel, covalent conjugation of CPMV-NB to the polymer network immobilizes particles during the photopolymerization, while in the blended hydrogel, particles remain free to migrate or become excluded to the surface as the network forms, resulting in surface localization and aggregation. The spatial distribution of CPMV particles has direct implications for release behavior. Particles embedded within the network experience greater steric confinement, resulting in degradation-controlled release. Conversely, surface-adhered particles are more accessible to the surrounding medium, which can lead to faster initial release dominated by diffusion.

The stability of the hydrogels in non-enzymatic environments was assessed by incubation in PBS and fetal bovine serum (FBS) at 37 °C with orbital agitation (100 rpm). PBS provides a physiologically relevant pH (pH 7.2) and osmotic pressure environment, whereas FBS represents a protein-rich fluidic environment. As shown in figure 3(b), the cylindrical hydrogels remained intact after 6 d in both PBS and FBS, showing only a slight, non-significant decrease in Young’s modulus (figure 3(c), left panel), while their dimensions were unchanged (figure 3(c), right panel).

To evaluate enzymatic degradation, collagenase was added to PBS and FBS, respectively. As shown in figure 3(d), hydrogels in PBS began to degrade within 15 min, exhibiting significant reductions in both Young’s modulus and diameter (figure 3(e)). After 30 min, the residual stress response of the hydrogel fell below the resolution limit of our mechanical tester setting (<0.604 $\mu $N). By 60 min, nearly all samples incubated in PBS had fully degraded, with only one sample showing minimal residue, which subsequently degraded upon prolonged incubation. In contrast, hydrogels in FBS remained intact throughout the experiment, as collagenase activity was inhibited by alpha-2-macroglobulin in the FBS. No significant changes in Young’s modulus or diameter were observed.

We further investigated the degradation of CPMV-NB hydrogels shaped as circular slabs (5 mm in diameter, 3 mm in height), with a geometry and CPMV loading chosen to mirror a murine subcutaneous or IP implant. Degradation was tested in PBS and 5% FBS to mimic physiological fluids encountered by implants. As shown in figure 3(f), the hydrogels remained intact for 7 d in both PBS and 5% FBS in the absence of enzymes. Upon addition of collagenase on day 7, hydrogels in PBS degraded rapidly, with visible degradation within 15 min, leaving only small residues after 4 h, and complete degradation by 8 h. In 5% FBS, degradation was slower due to partial inhibition of collagenase by alpha-2-macroglobulin; nevertheless, a slight but visible reduction in implant size was observed after one day of incubation. Together, these results demonstrate that the hydrogels remain stable under physiologically relevant non-enzymatic conditions but undergo controlled degradation in enzymatic environments, with rates dependent on enzyme activity.

**Figure 4.**
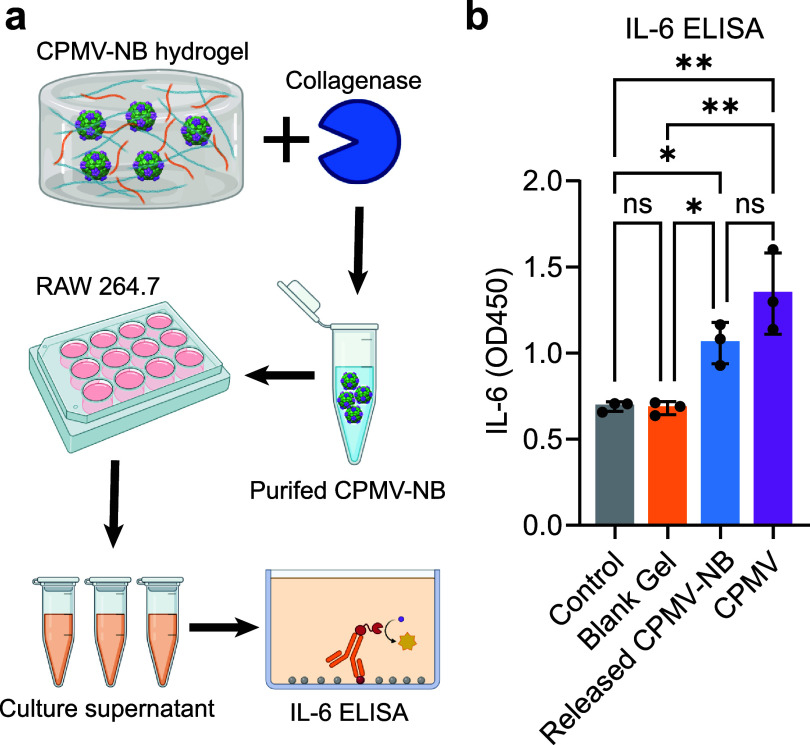
IL-6 ELISA to confirm the immunogenic function of the released CPMV-NB post collagenase digestion. (a) Scheme of the IL-6 ELISA experiment. (b) Result of the IL-6 ELISA experiment. *n* = 3 independent experiments; data are expressed as mean ± SD. Statistical significance was determined by ordinary one-way ANOVA. Part of (a) is generated in BioRender. Created in BioRender. Steinmetz, N. (2026) https://BioRender.com/z9q330n.

Lastly, we investigated whether CPMV-NB released via collagenase digestion retained its immunogenic activity. For POC, we used RAW264.7 macrophages and measured IL-6 cytokine secretion [35]. We first bioprinted CPMV-NB hydrogels, which were then incubated in 1 ml PBS at 37 °C for 6 d to remove unbound CPMV-NB by diffusion, which was followed by collagenase digestion. Collagenase alone induced IL-6 secretion in our preliminary experiment, so CPMV-NB was first purified from the digestion samples prior to cell treatment to remove hydrogel fragments and free collagenase. 50 *µ*g purified CPMV-NB, CPMV, and controls were then separately incubated with RAW264.7 macrophages, and released IL-6 in the conditioned media was measured by ELISA assay (figure 4(a)). As demonstrated by figure 4(b), released CPMV-NB maintained its immunogenicity to induce IL-6 secretion following collagenase triggered release from hydrogels comparable to naive CPMV (figure 4(b)).

In conclusion, we developed a proof-of-concept collagenase-responsive hydrogel system using DLP bioprinting technology to sufficiently load and retain a biologic immunotherapy candidate, namely CPMV nanoparticles. Upon collagenase treatment, the hydrogel released the norbornene functionalized CPMV (CPMV-NB). Characterization confirmed hydrogel stability and the structural integrity and immunostimulatory function of released CPMV-NB comparable to naive CPMV. We note that this is a POC study to demonstrate controlled delivery of CPMV from the hydrogel depot. Future work must address detailed biological assays to confirm that released CPMV-NB have suitable immunomodulatory properties to induce potent anti-tumor efficacy *in vivo*. In addition to testing in animal models, there is a need to develop advanced 3D tissue models and those that incorporate human tissues to address government priorities to reduce animal testing and prioritize human-based research. There also is room for optimization of the design: the cleavage peptide sequence could be tailored to the TME targeting specific enzymes matched to a specific tumor type. The release kinetics must be carefully evaluated *in vivo, ex vivo* or *in vitro* models that mimic the TME. From an engineering perspective, the collagenase-cleavable peptide can be readily substituted with peptides or linkers responsive to other enzymes [36]; similarly, other immunomodulatory or therapeutic biologic cargos and nanoparticles could be incorporated through norbornene functionalization—the developed system offers a plug-and-play technology. This platform thus establishes a foundation to develop a broad range of hydrogel-based depots capable of releasing their cargos into TME in response to enzymes secreted by tumor cells, fibroblasts, and/or immunosuppressive cancer cells [37] to prime an anti-tumor (immune) responses.

## Data Availability

All data that support the findings of this study are included within the main article and supplementary files. Raw data from this study are available upon reasonable request from the authors. Supplementary Information available at http://doi.org/10.1088/1758-5090/ae37de/data1.

## Supplementary information

The supplementary information includes Materials and Methods, Characterization of CPMV, and various CPMV-NB conjugations and NuPage gel densitometry analysis to quantify released CPMV-NB through collagenase digestion.
